# Array comparative genomic hybridisation in haematological malignancies: A comprehensive review

**DOI:** 10.4102/ajlm.v15i1.3117

**Published:** 2026-07-21

**Authors:** Mona Mohammed Hashim Ellaithi, Hussam Ali Osman

**Affiliations:** 1 Faculty of Medical Laboratory Sciences, Al-Neelain University, Khartoum, Sudan; 2 Faculty of Medical and Health Sciences, Liwa University, Abu Dhabi, United Arab Emirates

**Keywords:** array CGH, haematologic malignancies, copy number variations, molecular cytogenetics, precision medicine, diagnostic genomics

## Abstract

**Background:**

Haematologic malignancies have diverse and complex genomic abnormalities, and the correct identification is essential for making the appropriate diagnosis, providing a prognosis, and planning treatment. Improved array comparative genomic hybridisation (array CGH) can provide high-resolution, genome-wide copy number variation detection, and overcomes conventional cytogenetic limitations.

**Aim:**

The aim of this study is to determine the role of array CGH in characterising genomic aberrations of haematologic malignancies, focusing on technical advantages, added diagnostic values, and implications for disease reclassification and precision medicine.

**Methods:**

A comprehensive literature search of PubMed, Embase, Web of Science, and Scopus was conducted to identify studies evaluating the diagnostic performance and clinical utility of array CGH in haematologic cancers.

**Results:**

Array CGH detects genomic alterations at kilobase-level resolution and reveals additional abnormalities in approximately 30% of cases with normal results by conventional cytogenetics. It improves molecular subtyping, identifies novel prognostic marker and, when combined with single nucleotide polymorphism arrays, enables detection of uniparental disomy and copy-neutral loss of heterozygosity, thereby enhancing diagnostic yield.

**Conclusion:**

Array CGH detects up to 90% of known genomic abnormalities in haematologic malignancies, and its integration with other genomic platforms will considerably enhance diagnostic precision and clinical care for haematopoietic neoplasms.

**What this study adds:**

This review emphasises the important role of array CGH’s greater sensitivity to clinically relevant copy number changes compared to routine cytogenetics. Clinical applications of array CGH support precision oncology, especially when combined with single nucleotide polymorphism array or next-generation sequencing technologies.

## Introduction

### Haematological malignancies

Haematological malignancies comprise a heterogeneous group of cancers originating from haematopoietic and lymphoid tissues, including leukaemia, lymphoma, and multiple myeloma. These disorders are characterised by clonal proliferation of abnormal blood cells that disrupt normal haematopoiesis and immune function.^1^ Leukaemias are classified as acute or chronic and arise from myeloid or lymphoid lineages, whereas lymphomas develop within lymphatic tissues and are broadly categorised as Hodgkin or non-Hodgkin types.^2^ Multiple myeloma originate from malignant plasma cells in the bone marrow and presents with distinct clinical manifestations.^3^

Globally, these malignancies impose a substantial epidemiological burden, with incidence influenced by age, genetic predisposition, and environmental exposures. Common clinical features include anaemia, recurrent infections, lymphadenopathy, and bone lesions.^4,5^ Their marked biological heterogeneity complicates diagnosis and prognostication, making molecular and cytogenetic abnormalities central to disease classification and risk stratification.^6,7^

### History of cytogenetic analysis in haematology

Cytogenetic analysis has long been integral to the diagnosis and classification of haematologic neoplasms. Conventional techniques, such as G-banded karyotyping and fluorescence in situ hybridisation, detect chromosomal abnormalities, including translocations, deletions, and duplications, which carry diagnostic and prognostic significance, exemplified by the Philadelphia chromosome in chronic myeloid leukaemia and characteristic translocations in acute lymphoblastic leukaemia.^8^

**FIGURE 1:**
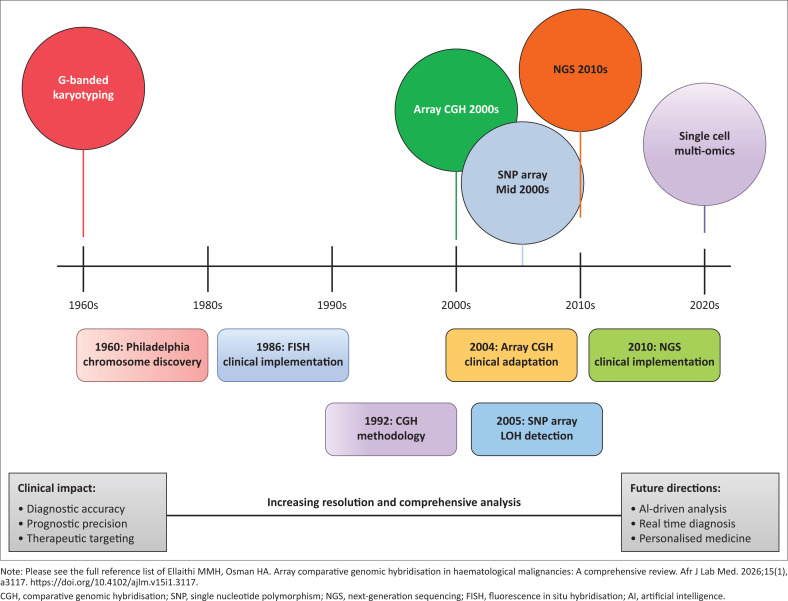
Evaluation of cytogenetic technologies in haematological malignancies. Timeline illustrating the evolution of cytogenetic and genomic technologies in haematological malignancies, from G-banded karyotyping and fluorescence in situ hybridisation to array CGH, single nucleotide polymorphism arrays, next-generation sequencing, and single-cell multi-omics. These advances reflect increasing genomic resolution and clinical integration, enabling improved diagnostic precision, risk stratification, and targeted therapeutic approaches.

However, these approaches have inherent limitations. Karyotyping requires actively dividing cells and has relatively low resolution, while fluorescence in situ hybridisation provides targeted analysis but lacks genome-wide coverage.^9^ Advances in molecular cytogenetics, particularly array comparative genomic hybridisation (array CGH), have addressed these constraints by enabling high-resolution, genome-wide detection of chromosomal imbalances^10^ (Figure 1).

### Introduction to array CGH

Array CGH is a high-resolution molecular cytogenetic technique that detects genome-wide copy number alterations (CNAs), including deletions, duplications, and amplifications, through competitive hybridisation of labelled test and reference DNA to microarray probes.^10,11^ Unlike conventional cytogenetic methods, array CGH does not require dividing cells, allowing analysis of non-dividing or archived samples and detection of copy number variations at kilobase-level resolution.^12^

**FIGURE 2:**
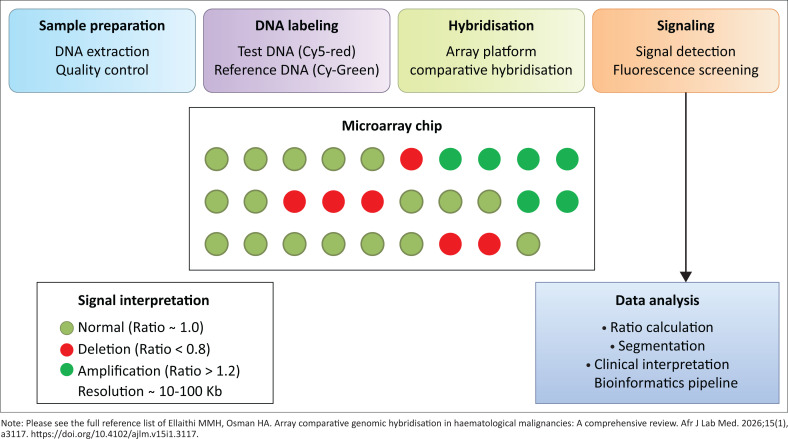
Array comparative genomic hybridisation workflow. This figure outlines the array comparative genomic hybridisation (array CGH) process, beginning with DNA extraction and labelling of test and reference samples, followed by co-hybridisation onto a microarray chip. Fluorescence signal detection enables identification of copy number changes. Data analysis includes ratio calculation and segmentation to classify genomic regions as normal, deleted, or amplified.

This technology has become widely applied in cancer research to characterise genomic complexity and identify alterations associated with tumour behaviour, therapeutic response, and clinical outcome.^13,14,15^ In haematological malignancies, array CGH refines cytogenetic evaluation and supports more precise diagnostic and prognostic assessment^12,16,17^ (Figure 2).

## Technical challenges and methodological advances

Array CGH platforms differ in probe type and density, directly influencing analytical resolution and sensitivity. High-density oligonucleotide arrays enable fine mapping of genomic aberrations at approximately 10 kb – 100 kb resolution,^18,19,20,21^ whereas bacterial artificial chromosome arrays provide robust hybridisation signals but lower resolution, typically several hundred kilobases. Disease-focused arrays enriched for genes frequently altered in haematologic malignancies further enhance clinical applicability. Incorporation of single nucleotide polymorphism probes allows detection of copy-neutral events such as loss of heterozygosity, thereby increasing diagnostic yield and identifying clinically relevant alterations.^22,23^

Integration of single nucleotide polymorphism arrays with array CGH enables identification of copy-neutral abnormalities, including uniparental disomy and loss of heterozygosity, which are not detectable by conventional CGH alone.^24,25,26^ Clinical studies demonstrate that this combined approach improves detection of cryptic genomic alterations in acute myeloid leukaemia, myelodysplastic syndromes, chronic lymphocytic leukaemia, and related disorders, refining diagnostic evaluation and risk stratification.^27,28^

Despite these advantages, technical and interpretative challenges remain. DNA quality, particularly in archived or treated samples, can affect performance. Tumour heterogeneity and subclonal diversity may reduce signal intensity and complicate CNA interpretation, necessitating advanced bioinformatic tools and specialised expertise.^14,29,30,31,32^ Balanced chromosomal rearrangements remain undetectable because they do not involve net DNA copy number changes, underscoring the need for complementary cytogenetic methods.^33,34^ Accurate interpretation also requires differentiation of pathogenic alterations from germline copy number polymorphisms and careful consideration of platform-specific thresholds.^35,36,37,38,39^

## Diagnostic utility in haematological malignancies

Array CGH enhances detection of CNAs compared with conventional cytogenetics. Approximately 90% of abnormalities identified by karyotyping and fluorescence in situ hybridisation are detected by array CGH, with an additional ~30% of clinically relevant CNAs identified in diagnostically important regions.^10,34,40^ Submicroscopic deletions and amplifications exceeding 20 Mb, undetectable by routine karyotyping, further increase diagnostic sensitivity and support more accurate classification.^12,41,42,43^

In precursor B-cell acute lymphoblastic leukaemia with *ETV6/RUNX1* t(12;21), array CGH reveals additional genomic abnormalities beyond the primary translocation, including recurrent losses (~77%) and gains (~23%) at loci such as 6q, 12p, 9p, 4q, and Xq, as well as microdeletions as small as 400 base pairs.^43,44,45,46^ These alterations frequently involve genes such as *RUNX1, CDKN2A, FHIT*, and *PAX5*, improving risk stratification and therapeutic decision-making.^47,48,49^

In chronic lymphocytic leukaemia/small lymphocytic lymphoma, deletion of 13q14 carries prognostic significance, but fluorescence in situ hybridisation does not define deletion extent or gene content.^50,51,52^ Array CGH combined with single nucleotide polymorphism arrays enables precise mapping of deletions up to 39 Mb and characterisation of genes including *TRIM13, miR-3613, KCNRG, DLEU2, miR-16-1, miR-15a, DLEU1*, and *RB1*.^51,53^ Distinguishing monoallelic from biallelic deletions and assessing loss of heterozygosity refines prognostic evaluation, particularly when integrated with *IGVH* mutation status^54,55^ (Table 1).

**TABLE 1:** Comparison of cytogenetic methods in haematological cancer diagnosis.

Parameter	Karyotyping	FISH	Array CGH	SNP array	NGS (WGS/WES)
Resolution	5 Mb – 10 Mb	100 Mb – 200 Mb	10 Kb – 100 Kb	10 Kb – 100 Kb	Single base pair
Genome coverage	Whole genome	Targeted region	Whole genome	Whole genome	Whole genome
Sample requirement	Dividing cells	Fresh and fixed tissue	Any source of DNA	Any source of DNA	Any source of DNA
Turnaround time (days)	3–7	1–2	2–3	2–3	5–14
Technical expertise	High	Moderate	High	High	Very high
**Detectable alterations**					
Balanced translocations	Yes	Yes	No	No	Yes
Deletions/duplications	Yes (> 5 Mb)	Yes (targeted)	Yes	Yes	Yes
Copy number alterations	Yes (large)	Yes (targeted)	Yes	Yes	Yes
Loss of heterozygosity	No	No	No	Yes	Yes
Point mutations	No	No	No	No	Yes
Structural variations	Yes (large)	Yes (targeted)	No	No	No
**Advantages**					
	Gold standard	Rapid results	High resolution	LOH detection	Comprehensive
	Balanced rearrangements	Targeted analysis	Genome-wide	Copy-neutral events	Mutation detection
	Clonal heterogeneity	Interphase analysis	Automated analysis	Automated analysis	Structural variants
**Limitations**					
	Low resolution	Limited coverage	No balanced events	No balanced events	High cost
	Requires dividing cells	Requires prior knowledge	Complex interpretation	Complex interpretation	Long turnaround
	Labour intensive	Limited multiplexing	Germline CNVs	Germline CNVs	Data complexity
**Clinical application**					
	First-line screening	Targeted confirmation	Comprehensive profiling	Prognostic markers	Precision medicine
	Complex karyotyping	MRD monitoring	Cryptic alterations	Risk stratification	Therapeutic targets
	Chromosome instability	Specific translocations	Specific translocations	CNV characterisation	Resistance mechanisms
Sensitivity	Low–Moderate	High (targeted)	High	High	Very high
Specificity	High	Very high	Moderate–High	Moderate–High	High
Standardisation	Established	Established	Developing	Developing	Emerging

Note: Please see the full reference list of Ellaithi MMH, Osman HA. Array comparative genomic hybridisation in haematological malignancies: A comprehensive review. Afr J Lab Med. 2026;15(1), a3117. https://doi.org/10.4102/ajlm.v15i1.3117.

FISH, fluorescence in situ hybridisation; CGH, comparative genomic hybridisation; SNP, single nucleotide polymorphism; NGS, next-generation sequencing; WGS, whole genome sequencing; WES, whole exome sequencing; CNV, copy number variation; LOH, loss of heterozygosity; MRD, minimal residual disease; Mb, megabase; Kb, kilobase.

## Prognostic and therapeutic implications

Array CGH has identified recurrent CNAs associated with treatment response and survival. In diffuse large B-cell lymphoma, gains at 2p16 and deletions at 17p13 (*TP53*) and 10q23.31 (*PTEN*) correlate with poor chemotherapy response and reduced survival.^47,56,57,58,59,60^ In acute myeloid leukaemia, detection of copy-neutral loss of heterozygosity using combined CGH/single nucleotide polymorphism arrays is associated with inferior relapse-free survival, emphasising the prognostic value of high-resolution genomic profiling^49,61,62,63,64,65,66,67^ (Table 2).

**TABLE 2:** Key array comparative genomic hybridisation findings in haematological cancer subtypes.

Cancer subtype	Common CNAs detected	Cryptic alterations	Clinical significance	Diagnostic yield	Prognostic
*ETV6/RUNX1* positive	del(6q), del(12p), del(9p)	Microdeletions (> 400 bp)	Risk stratification	77% losses, 23% gains	*CDKN2A, PAX5* alterations
Hyperdiploidy	dup(4q), dup(Xq)	*RUNX1, FHIT* deletions	Treatment selection	> 90% detection rate	Better prognosis
*BCR-ABL1*-like	del(7p), del(9p21)	*IKZF1* deletions	High-risk identification	85% aberrant cases	Poor outcome predictor
**Chronic lymphocytic leukaemia**					
*13q14* deletions	del(13q14.2-q14.3)	Size: 0.3 Mb – 39 Mb range	Prognostic stratification	50% of patients	Mono vs. biallelic impact
Complex alterations	del(11q), del(17p)	LOH events	Treatment resistance	Enhanced detection	*TP53* pathway disruption
Trisomy 12	+12, +19	Subclonal populations	Disease heterogeneity	Superior to FISH	Intermediate prognosis
**Diffuse large B-cell lymphoma**					
GCB subtype	del(1p36), amp(2p16)	*BCL6* rearrangements	Molecular subtyping	30% additional CNAs	Response to R CHOP
ABC subtype	del(6q), amp(3q)	*MYC* amplifications	Aggressive variants	Enhanced classification	Poor survival marker
Primary mediastinal	amp(9p24), amp(2p16)	PD-L1/PD-L2 gains	Therapeutic targeting	Novel alterations	Immunotherapy response
**Multiple myeloma**					
Hyperdiploid	+3,5,7,9,11,15,19,21	Chromothripsis events	Risk stratification	Complex karyotypes	Standard risk
Non-hyperdiploid	del(13q), del(17p)	*TP53* deletions	High-risk disease	Cryptic deletions	Poor prognosis
*1q21* amplification	amp(1q21-q23)	*CKS1B, MCL1* gains	Treatment resistance	Subclonal detection	Adverse outcome
**Peripheral T-cell lymphoma**					
PTCL-NOS	+7p, +7q, del(9p21.3)	*CDKN2A/B* deletions	Molecular classification	Recurrent patterns	Poor prognosis markers
ATLL comparison	Similar profiles to PTCL	Shared genetic signatures	Diagnostic refinement	Overlapping alterations	Biological insights
Anaplastic large cell	+7, del(6q)	ALK-negative variants	Subtype distinction	Additional CNAs	Treatment stratification
**Acute myeloid leukaemia**					
Complex karyotype	Multiple CNAs	Chromothripsis	Risk assessment	Superior detection	Very poor prognosis
Normal karyotype	Cryptic deletions	Copy-neutral LOH	Hidden alterations	20-30% additional	Intermediate risk refinement
Core-binding factor	del(9q), +8	*KIT* amplifications	Additional lesions	Cooperative events	Treatment modification
**Myelodysplastic syndromes**					
del(5q) syndrome	del(5q31-q33)	Size variations	Lenalidomide response	Precise mapping	Treatment predictor
Complex alterations	Multiple CNAs	*TP53* pathway	Risk stratification	Enhanced detection	Poor survival
Therapy-related	del(7q), del(5q)	Chromothripsis	Secondary malignancies	Characteristic patterns	Very poor prognosis
**Chronic myelomonocytic leukaemia**					
CMML-1/2	+8, del(20q)	*RAS* pathway alterations	Molecular subtyping	Novel fusions	Disease progression
Secondary mutations	*RUNX1* alterations	*USP16–RUNX1* fusion	Cooperative events	Cryptic inversions	Pathogenesis insights

Note: Please see the full reference list of Ellaithi MMH, Osman HA. Array comparative genomic hybridisation in haematological malignancies: A comprehensive review. Afr J Lab Med. 2026;15(1), a3117. https://doi.org/10.4102/ajlm.v15i1.3117.

CNA, copy number alteration; del, deletion; dup, duplication; amp, amplification; LOH, loss of heterozygosity; Mb, megabase; bp, base pair; GCB, germinal centre B-cell-like; ABC, activated B-cell-like; PTCL-NOS, peripheral T-cell lymphoma–Not otherwise specified; ATLL, adult T-cell leukaemia/lymphoma; CMML, chronic myelomonocytic leukaemia; CNV, copy number variation; R-CHOP, Rituximab, cyclophosphamide, doxorubicin, vincristine, and prednisone), ALK, anaplastic lymphoma kinase; PD-L1, programmed death-ligand 1; PD-L2, programmed death-ligand 2.

High-resolution genomic analysis also facilitates identification of actionable alterations linked to drug resistance or disease aggressiveness, supporting individualised treatment strategies when integrated with clinical and molecular data.^10,16,68,69^ However, genomic complexity and subclonal architecture may influence prognostic interpretation.^70,71^

### Molecular subtyping of haematological cancers

Array CGH contributes to molecular subclassification across haematologic malignancies. In diffuse large B-cell lymphoma, which accounts for over 30% of adult lymphomas and exhibits substantial heterogeneity,^72^ recurrent genomic gains and losses affecting ≥ 20% of cases define subtype-specific genomic signatures correlated with gene expression profiles and clinical outcomes.^72,73,74,75,76^

In peripheral T cell lymphoma, unspecified, high-density array CGH identifies recurrent gains at 7p and 7q and deletions at 9p21.3, associated with adverse prognosis.^77,78^ Comparative profiling with adult T-cell leukaemia/lymphoma reveals shared genomic features that support refined classification.^78^

Additional contributions include genomic characterisation of myelodysplastic syndromes, myeloproliferative neoplasms, and chronic myelomonocytic leukaemia.^79,80,81,82,83^ In chronic myelomonocytic leukaemia, recurrent abnormalities such as trisomy 8, deletion 20q, *RAS* and *RUNX1* mutations, and cryptic inversions including *USP16-RUNX1* fusion illustrate genomic complexity and disease heterogeneity.^17,49,84,85,86,87^

## Discussion

### Comparative analysis with other technologies

Array CGH provides genome-wide, high-resolution detection of CNAs, surpassing conventional cytogenetics for submicroscopic abnormalities.^10,12,16^ While balanced rearrangements require complementary approaches, combined use of cytogenetic and molecular technologies increases diagnostic yield and refines molecular subtyping.

Integration with next-generation sequencing enables detection of point mutations, small insertions and/or deletions, and structural variants, providing a comprehensive genomic profile that improves disease classification and therapeutic targeting.^88,89,90,91,92,93,94,95,96^ Emerging developments, including single-cell array CGH, enhanced probe density, and advanced bioinformatic algorithms, further improve detection of clonal evolution and complex genomic architecture.^97,98,99,100,101,102^

### Clinical implementation and quality assurance

International cytogenomic guidelines recommend array CGH as a complementary diagnostic tool, emphasising assay validation, quality control, and standardised interpretation.^12,103,104,105^ Successful implementation requires integration with clinical, pathological, and molecular data to ensure diagnostic reliability.

Adoption may be limited by infrastructure requirements, costs, and the need for specialised expertise.^12^ Nevertheless, clinical studies in paediatric leukaemia and chronic lymphocytic leukaemia demonstrate that array CGH identifies additional abnormalities beyond conventional cytogenetics, improving molecular characterisation and prognostic stratification.^44,106,107^

### Biological insights and research applications

Array CGH has expanded understanding of tumour biology by identifying subclonal CNAs and tracking clonal evolution, thereby elucidating mechanisms of relapse and therapeutic resistance.^68,104,108,109^ It has also uncovered cryptic deletions, novel CNAs, and gene fusions affecting oncogenes, tumour suppressors, and regulatory RNAs.^10,110,111,112,113,114,115^ These discoveries contribute to biomarker identification and the development of targeted therapeutic strategies, reinforcing their role in translational haematology research.^116^

### Limitations and future directions

Array CGH cannot detect balanced rearrangements or very small sequence variants, necessitating complementary molecular approaches.^117^ Integration with sequencing-based, transcriptomic, and proteomic analyses enables more comprehensive genomic characterisation.^78,118^ Continued improvements in probe density, hybridisation chemistry, computational algorithms, and single-cell applications are expected to enhance sensitivity and analytical precision.^119,120^

### Impact on personalised medicine

Incorporation of array CGH into clinical practice enables identification of clinically actionable genomic alterations and refined prognostic stratification, supporting individualised therapeutic strategies and improved patient management.^12,121^

### Conclusion

Array CGH has substantially advanced cytogenetic evaluation of haematologic malignancies by enabling high-resolution, genome-wide detection of DNA CNAs. When integrated with conventional cytogenetics and sequencing technologies, it enhances diagnostic accuracy, prognostic assessment, molecular subclassification, and personalised treatment planning. Continued technological refinement, standardisation, and integrative genomic strategies will further consolidate its role in precision haematologic oncology.
